# Medical Revolutions

**Published:** 1915-08

**Authors:** T. D. Crothers

**Affiliations:** Hartford, Conn.


### EDITORIAL DEPARTMENT.

MRS. F. E. DANIEL, Publisher and Managing Editor.
DR. R. H. L. BIBB, Associate Editor.

CONTRIBUTING EDITORS:

Dr. H. Sheridan Baketel, New York, editor *Medical Times:* Dr. M. H. Boerner* Austin, Texas; Dr. G. Henri Bogart, Paris, Ill.; Dr. M. M. Carrick, Dallas, Texas; Dr. Edward H. Carey, Dean Medical Department, Baylor University, Dallas, Texas; Dr. W. L. Crosthwaite, Waco, Texas; Dr. T. D. Crothers, Hartford, Conn.; Dr. H. W. Cummings, Hearne, Texas; Dr. Oscar Dowling, New Orleans, President Louisiana State Board of Health; Havelock Ellis, M. D., London, Eng.; Dr. May Agnes Hopkins, Dallas, Texas; Dr. A. W. Fly, Galveston, Texas; Dr. G. B. Foscue, Waco, Texas; Dr. E. S. Goodhue, Holualoa, Kona, Hawaii; Dr. M. B. Grace, Seguin, Texas; Dr. Joe Gilbert, Austin, Texas; Dr. Charles H. Hughes, St. Louis, editor *Alienist and Neurologist*; Dr. James G. Kiernan, Chicago; Dr. George H. Lee, Galveston, Texas; Dr. John T. Moore, Houston, Texas; Dr. Frank Paschai, San Antonio, Texas; Dr. John Preston, Austin, Texas, Superintendent State Lunatic Asylum; Dr. G. Wilse Robinson, Kansas City, Mo.; Dr. Bacon Saunders, Fort Worth, Texas; Dr. Allen J. Smith, Philadelphia, Medical Department, University of Pennsylvania; Dr. Z. T. Scott, Austin, Texas; Dr. Ralph Steiner, Austin, Texas, President Texas State Board of Health; Dr. Walter S. Sutton, Kansas City, Mo.; Dr. A. E. Sweatland, Nacogdoches, Texas; Dr. John S. Turner, Dallas, Texas; Dr. Charles Venable, San Antonio, Texas.

### Medical Revolutions.

BY T. D. CROTHERS, M. D., HARTFORD, CONN.

This was in part the title of a very distinguished man's address on a great occasion. He seemed to think that the discovery of ether and the bacterial origin of disease, and some other things of lesser note, were the great epoch-making events of the century. The way in which he grouped the facts showed an internal confusion of mind that was more or less startling. His oration was a midnight product, evidently, and did not represent the man at his best.

The real medical revolutions of the day were evidently unknown and are obscure and unrecognized, to a large degree, by the medical public. Notwithstanding the somewhat extravagant efforts made to raise the standard of education and develop a higher kind of training, and a better class of physicians, the profession is involved in a fog bank of theories and credulities, based on traditions and actual superstitions, which have come down from the past and still retain their hold in medical circles.

One of these traditions is that a medical man is made by scholastic and scientific training; that if he has passed through all the schools, and filled all the requirements, he represents the modern physician. Another is there are certain fixed principles of science which must be regarded as absolute, and their attainment and practical realization means progress and development to be sought after.

A third delusion is that the teachings of the fathers in colleges and text-books are the great ideals from which to measure everything of the present. In one particular, the credulity of the present stands out very markedly; that is, our present requirements of entrance to the study of medicine ignore the physical fitness and capacity of the man.

Today it is possible for anyone to take up the study of medicine and become a physician. He may be halt, lame and blind, and possess of mental and physical feebleness; he may be a moral paretic, a paranoiac or degenerate, and even epileptic, yet, if he has secured a degree from a literary college or high school, he can enter a medical college, pass the examining board and develop a phonographic memory, and answer all questions, and meet the requirements of such boards, and go out with a diploma, entitling him to practice medicine in every degree possible. No question is ever raised of his physical and mental defects. As a result the medical profession does not represent the type of men it should.

Circumstances and surroundings may hold them up to a standard which they could not attain otherwise, but, as in other professions, they are by no means representative as teachers of hygiene and higher physical and mental health.

In almost every medical circle there are weaklings who are not only a disgrace but a menace to the welfare of the people. The remedy is to make the requirements to enter the medical profession turn on the physical capacity, and follow the government plan of refusing to train defective and physically imperfect young men, and insist that they be the highest physical types before they begin to study, and that the training they receive in the medical colleges shall concern the entire organism, both physical and mental.

Here is where a medical revolution is urgently called for. The profession should demand a degree of physical attainment and idealism from every young man. When he has entered the profession, he should exemplify in his own life and conduct the best types of a student of the great problems of nature. It is not the school or college; it is the man. If he possesses good average ability and good health, and a fair normal training of both mind and body, there is little danger of him falling into disreputable ways. Such a man is never a quack in the sense of being deceptive, dishonest and tricky in his conduct and work.

Revolutions in medicine should be, not the discovery of new facts, but finding men who are capable of appreciating these facts and their relation, and putting them to the service of mankind.

Many doctors show their incapacity and weakness in their failure to apply these facts in their own lives. They are emphatic in their advice to others, but give no evidence of using it themselves.

A diseased and sickly doctor is an advertisement of his failure. If this learned professor had called attention to this fact, as a revolution of most practical importance, he would have opened a new realm of medicine with vast possibilities yet to be attained.

The revolution should develop scholars, students, reasonable, rational, earnest thinkers; men who will point out fields of preventive medicine, who will show how psychical therapeutics can be put to service and contribute towards the development of life as much so as physical therapeutics; men who will show how longevity can be increased, disease diminished, and how life can be made broader and freer from difficulties and failures.

It is literally a grand effort to develop the divinity of man and to bring him into full co-operation with the physical and psychical laws of nature. The revolution should raise the physician to a plain above the clergyman and men of all other occupations. There is no idealism in this. It is becoming more and more practical every day. Each physician should study to make himself the typical man of his neighborhood and circle of the best health, the best judgment, living the purest and best life, and thus become the great priest of Hygeia.

